# MicroRNA-379-5p is associated with biochemical premature ovarian insufficiency through *PARP1* and *XRCC6*

**DOI:** 10.1038/s41419-017-0163-8

**Published:** 2018-01-24

**Authors:** Yujie Dang, Xiaoyan Wang, Yajing Hao, Xinyue Zhang, Shidou Zhao, Jinlong Ma, Yingying Qin, Zi-Jiang Chen

**Affiliations:** 1 Renji Hospital, Shanghai Jiao Tong University School of Medicine, Shanghai, 200001 China; 2 Center for Reproductive Medicine, Shandong Provincial Hospital Affiliated to Shandong University, Jinan, 250021 China; 3 National Research Center for Assisted Reproductive Technology and Reproductive Genetics, Jinan, 250001 China; 4 The Key Laboratory of Reproductive Endocrinology (Shandong University), Ministry of Education, Jinan, 250001 China; 5 Shandong Provincial Key Laboratory of Reproductive Medicine, Jinan, 250001 China; 6 Key Laboratory of RNA Biology, Institute of Biophysics, Chinese Academy of Sciences, Beijing, 100101 China; 7 Beijing Key Laboratory of Noncoding RNA, Institute of Biophysics, Chinese Academy of Sciences, Beijing, 100101 China; 8 University of Chinese Academy of Sciences, Beijing, 100049 China; 9 Center for Reproductive Medicine, Renji Hospital, School of Medicine, Shanghai Jiao Tong University, Shanghai, 200135 China; 10 Shanghai Key Laboratory for Assisted Reproduction and Reproductive Genetics, Shanghai, 200135 China

## Abstract

Premature ovarian insufficiency (POI) imposes great challenges on women’s fertility and lifelong health. POI is highly heterogeneous and encompasses occult, biochemical, and overt stages. MicroRNAs (miRNAs) are negative regulators of gene expression, whose roles in physiology and diseases like cancers and neurological disorders have been recognized, but little is known about the miRNAs profile and functional relevance in biochemical POI (bPOI). In this study, the expression of miRNAs and mRNAs in granulosa cells (GCs) of bPOI women was determined by two microarrays, respectively. MiR-379-5p, *PARP1*, and *XRCC6* were differentially expressed in GCs of bPOI as revealed by microarrays. Subsequently, functional studies demonstrated that miR-379-5p overexpression inhibited granulosa cell proliferation and attenuated DNA repair efficiency. Furthermore, both *PARP1* and *XRCC6* showed lower levels in GCs from patients with bPOI and were identified as executives of miR-379-5p. Therefore, our data first uncovered potentially pathogenic miR-379-5p and two novel targets *PARP1* and *XRCC6* in bPOI, which corroborated the significance of DNA repair for POI, and brought up an epigenetic explanation for the disease.

## Introduction

Premature ovarian insufficiency (POI) is a hypergonadotropic disorder, which imposes great challenges on women’s fertility and lifelong health. Diagnosis is confirmed by amenorrhea for at least 4 months and elevated serum FSH levels (>25 IU/l) before 40 years of age^1,2^. The prevalence of POI varies by ethnicity. A latest study shows that 2.8% of the Chinese women are affected with POI^3^. Ovarian function decline is thought to be a continuous spectrum, three stages of POI have been described, including occult (subfertility or incipient ovarian insufficiency), biochemical insufficiency (raised concentrations of FSH, also known as transitional ovarian failure), and eventually overt (also termed as premature ovarian failure, POF [MIM 311360]) phages^4–6^. As the disorder might take several years to proceed, early detection and intervention for high-risk women may be beneficial to rescue fertility.

POI is a heterogeneous disease and two hypotheses of mechanism have long been recognized: inadequate primordial follicle pool, or accelerated depletion of oocytes and follicles^7,8^. Animal models suggest that dysfunction of granulosa cells is closely associated with follicle atresia, and microRNAs may play important roles during this process^9–13^. MicroRNA (miRNA) is a class of small regulatory noncoding RNA, fine-tuning gene expression post transcriptionally through mRNA degradation and inhibition of translation initiation^14^. It has been shown that disruption of miRNAs may contribute to physiological processes and human diseases^15^, likewise polymorphisms and abnormal expression of miRNAs were observed associated with POI^16–21^. However, given limited samples and functional evidences, miRNAs in granulosa cells (GCs) of biochemical POI (bPOI) individuals have yet to be determined.

Biochemical POI, in which stage women have regular menses but elevated FSH levels and reduced fertility, is the prior stage before follicle exhaustion completely. Here we performed miRNA and mRNA microarray in GCs from bPOI to uncover altered profiles of miRNAs and genes, then we combined bioinformatics analysis, quantitative reverse transcription (qRT)-PCR, and in vitro experiments to investigate the roles of miRNAs in bPOI. Results identified significantly upregulated miR-379-5p in GCs from bPOI patients, which suppressed cell proliferation and impaired DNA repair function through directly targeting poly ADP-ribose polymerase1 (*PARP1*) and X-ray repair complementing defective repair in Chinese hamster cells 6 (*XRCC6*), which were downregulated in GCs from the same cohort of cases.

## Results

### Clinical characteristics of all participants

The current study comprised 33 patients with biochemical POI and 30 age- and body mass index (BMI)-matched reproductive aged women with normal ovarian reserve. The bPOI patients undergoing in vitro fertilization/intracytoplasmic sperm injection and embryo transfer (IVF/ICSI-ET) were recruited from the Center for Reproductive Medicine, Shandong University. Inclusion criteria included (i) basal FSH (on days 2–4 of menstrual cycle) >10 mIU/ml; (ii) prior to 35 years of age; (iii) bilateral ovarian antral follicle count (AFC) <10. Thirty women with regular menstrual cycles and normal FSH level (<10 mIU/ml), who sought for infertility treatment due to tubal obstruction or male factors were enrolled as controls. Women with recurrent spontaneous abortion, chromosomal abnormality, history of chemo- or radio-therapy, ovarian surgery, or autoimmune diseases were excluded. The average age at recruitment was 30.55 ± 3.50 and 29.20 ± 3.56, respectively. Patients with bPOI exhibited typical endocrine profiles with moderately elevated bFSH levels, and dramatically less numbers of AFC and oocytes retrieved. Characteristics are summarized in Table 1.

**Table 1:** Clinical characteristics of patients with biochemical POI and controls

Variable	bPOI (*n* = 33)	Control (*n* = 30)	*P* value
Baseline characteristics			
Age (y)	30.55 ± 3.50	29.20 ± 3.56	0.137^a^
BMI (kg/m^2^)	21.41 (19.35, 25.24)	21.71 (20.01, 22.88)	0.995^b^
Basal FSH (IU/l)	12.50 (11.92, 15.98)	6.05 ± 1.27	<0.001^b^
Basal LH (IU/l)	5.05 (3.76, 7.81)	5.12 ± 1.59	0.433^b^
Basal FSH/LH	2.74 ± 0.83	1.25 ± 0.32	<0.001^a^
AFC	6.65 ± 2.97	14.41 ± 4.51	<0.001^a^
Basal E2 (pg/ml)	30.10 (15.73, 39.60)	30.70 (25.93, 42.97)	0.331^b^
AMH (ng/ml)	0.45 (0.26, 0.72)	2.90 (2.09, 5.66)	<0.001^b^
IVF treatment cycle parameters			
Number of follicles on day of HCG (≥14 mm)	3.00 (2.00, 4.00)	10.00 (8.00, 12.00)	<0.001^b^
Oocytes retrieved	3.00 (2.00, 4.00)	10.63 ± 3.40	<0.001^b^

Data are presented as mean ±SD or median (interquartile range (IQR)) based on distribution

*BMI* body mass index, AFC antral follicle count; AMH anti-Mullerian hormone; IVF in vitro fertilization

^a^Student's *t* test

^b^Mann–Whitney *U*-test

### MiR-379-5p, *PARP1*, and *XRCC6* are differentially expressed in GCs of bPOI as revealed by microarray

**Fig. 1:**
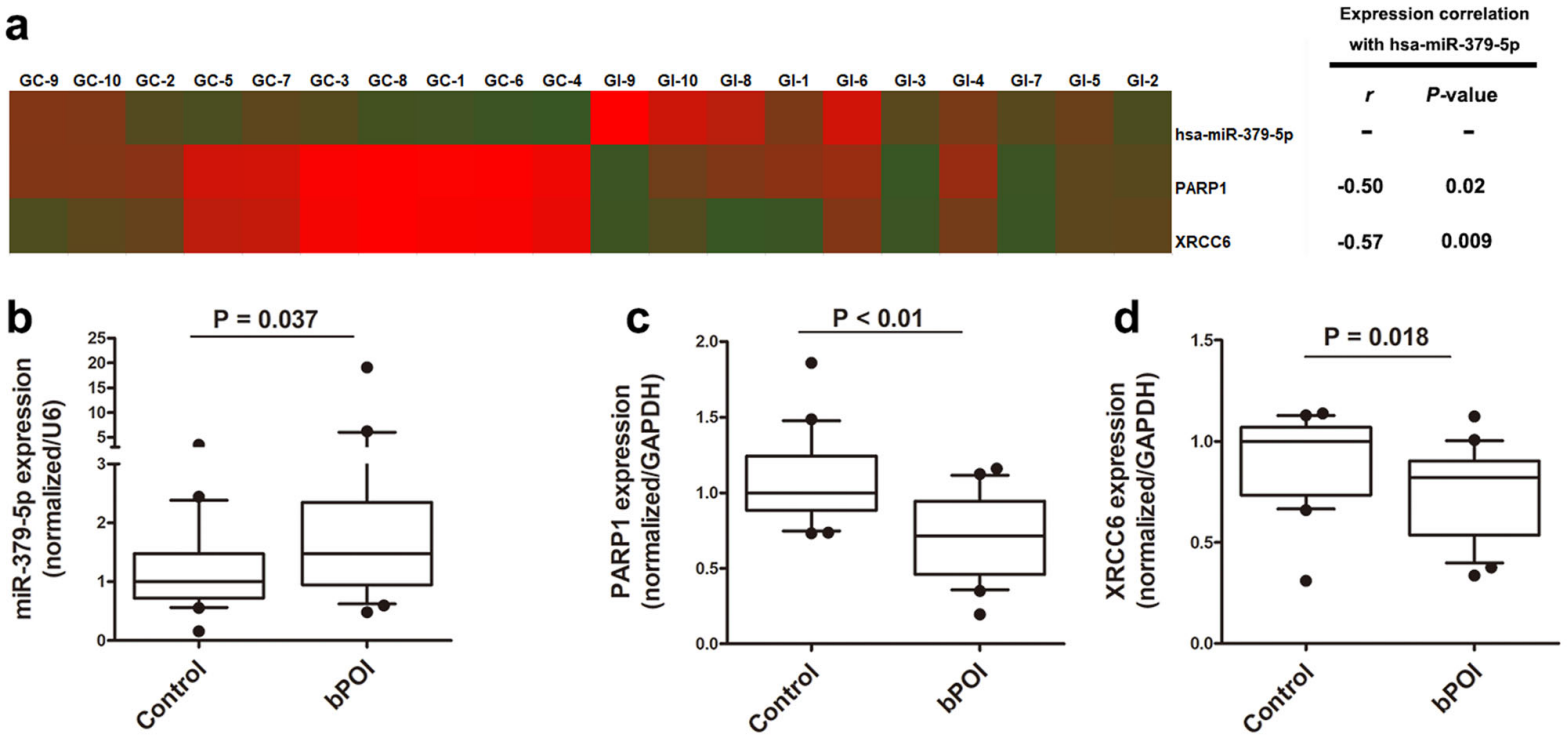
MiR-379-5p, *PARP1*, and *XRCC6* are differentially expressed in GCs of bPOI as revealed by microarray **a** Heat map of miR-379-5p, *PARP1*, and *XRCC6* based on microarray data. Each column represents a sample; red and green indicates up- and downregulation, respectively; r correlation coefficient. **b**,** c**,** d** Quantitative PCR (qPCR) analysis in GCs from women with and without bPOI shows that miR-379-5p increases (**b**), while *PARP1* and *XRCC6* decrease in bPOI patients (**c**,** d**). Two-tailed Mann–Whitney *U*-test

To investigate the expression profiles of miRNAs and mRNAs in GCs of bPOI patients, we first examined the change.s of miRNAs and mRNAs expression using microarrays (Exiqon miRCURY™ microarray LNA v.18.0 and Arraystar microarray v3.0) of GCs from ten bPOI and ten age- and BMI-matched normal control women. Seventy-five miRNAs (fold change (FC) >2, *P* < 0.05) and 3782 mRNAs (FC >2, *P* < 0.05; data not shown) were identified and differentially expressed between bPOI patients and control women. Of the 75 miRNAs changed in bPOI, 30 were upregulated and 45 were downregulated. Exiqon array data are available in the Gene Expression Omnibus database under accession number GSE100238. To systematically evaluate how the differentially expressed miRNAs in GCs influence gene expression and cellular activities, a miRNA–mRNA conjoint analysis was conducted and generated a bipartite miRNA/mRNA regulatory network. Then, the Gene Ontology analysis was performed to classify the putative target genes into different functional groups, which revealed an appealing biological process category of DNA repair. Hsa-miR-379-5p was one of the most upregulated miRNAs uncovered by the microarray, which exhibited a significantly negative correlation with DNA repair genes *PARP1* and *XRCC6* (Fig. 1a).

MiR-379-5p is located at a highly conserved imprinted DLK1-DIO3 genomic region on 14q32.31, which shows great developmental importance and signatures in schizophrenia and metabolic disease^22–25^. Mature miR-379-5p harbors a conserved seed sequence. Disruption of miR-379-5p has been reported in cancers, but little is known about its roles in endocrinopathy^26–28^. Both *PARP1* and *XRCC6* were putative targets of miR-379-5p and downregulated in bPOI through the mRNA microarray (Fig. 1a). Numerous mutations or polymorphisms in DNA repair genes have been identified causative or associated with reproductive ageing and POI, underling the importance of DNA repair function in human fecundity^29–38^. Consequently, DNA repair-associated genes, *PARP1* and *XRCC6,* were chosen as targets of miR-379-5p for further study.

To validate the microarray results, we analyzed miRNAs and mRNAs expression using qRT-PCR in an independent 43 GCs that were from bPOI (*n* = 23) and controls (*n* = 20). Consistent with microarray results, qRT-PCR showed that miR-379-5p increased (FC = 1.48, *P* = 0.037; Fig. 1b), while *PARP1* and *XRCC6* decreased significantly in the GCs from patients with bPOI (FC_*PARP1*_ = 0.72, P_*PARP1*_ < 0.01, Fig. 1c; FC_*XRCC6*_ = 0.82, P_*XRCC6*_ = 0.018, Fig. 1d).

### *PARP1* and *XRCC6* are novel targets of miR-379-5p

**Fig. 2:**
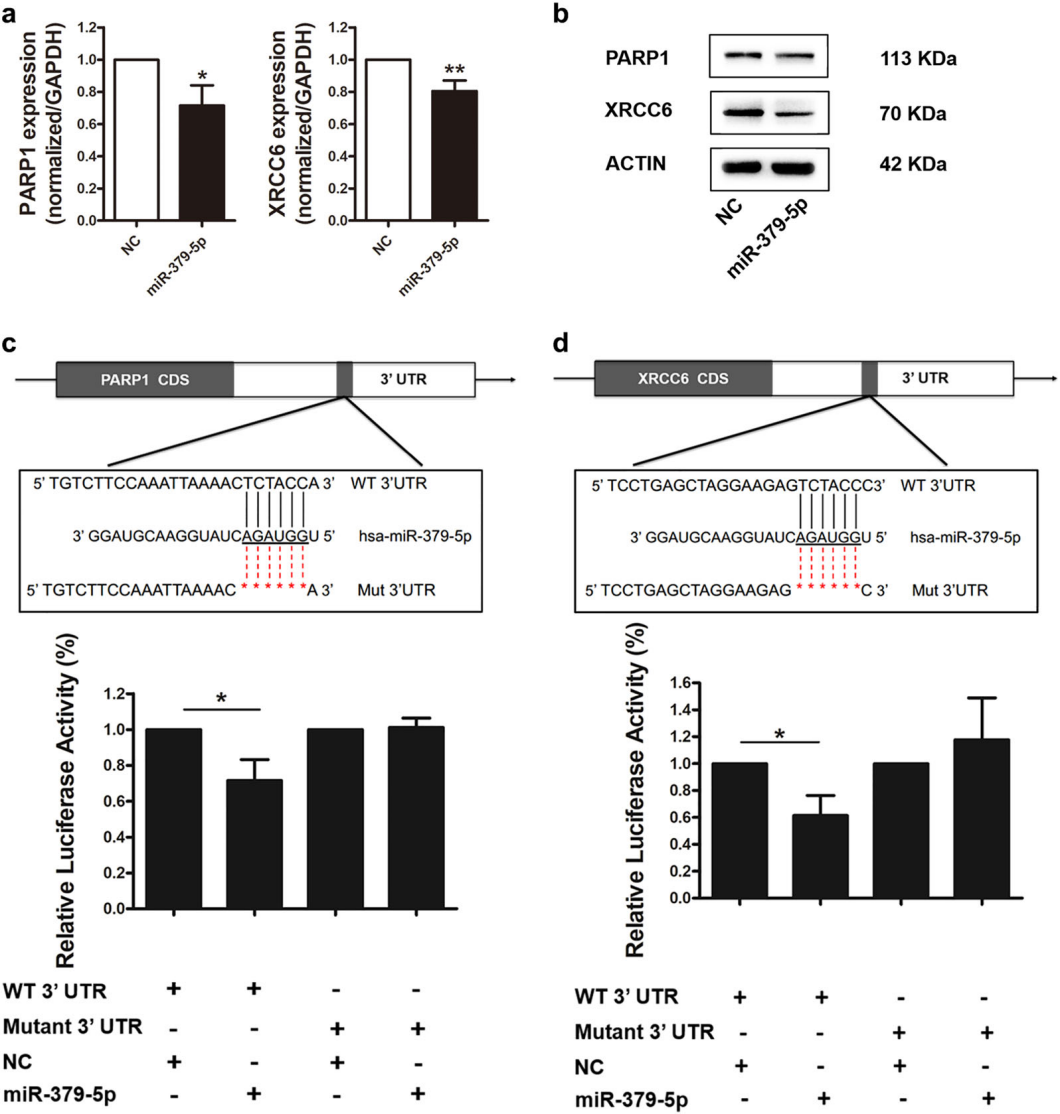
*PARP1* and *XRCC6* are novel targets of miR-379-5p **a**,** b** qRT-PCR and western blot analyses indicate that *PARP1* and *XRCC6* mRNA (**a**) and protein (**b**) levels are significantly reduced by forced expression of miR-379-5p in KGN cells. **c**,** d** Predicted target sites and relevant mutation sequences are shown. Luciferase assay demonstrates that overexpression of miR-379-5p leads to a decrease in *PARP1* and *XRCC6* with wild-type 3′-UTRs, while luciferase activity levels of mutated 3′-UTRs unchanged. Asterisk (*) indicates a significant difference. *P < 0.05, two-tailed Student's *t* test

*PARP1* and *XRCC6* are potential targets of miR-379-5p through in silico analyses. To verify the regulation of miR-379-5p on target genes, qRT-PCR and western blot were performed and indicated that *PARP1* and *XRCC6* mRNA and protein levels were significantly reduced by forced expression of miR-379-5p in KGN cells (Fig. 2a, b). To confirm the predicted binding sites of miR-379-5p in the 3′-UTRs of *PARP1* and *XRCC6*, we cloned the wild type and mutated 3′-UTRs of *PARP1* and *XRCC6* in a luciferase reporter vector. Luciferase assay demonstrated that overexpression of miR-379-5p led to a considerable decrease in *PARP1* and *XRCC6* with wild-type 3′-UTRs, while luciferase activity levels of mutated 3′-UTRs unchanged (Fig. 2c, d). These results suggested that *PARP1* and *XRCC6* were novel targets of miR-379-5p.

### Forced expression of miR-379-5p leads to proliferation inhibition and DNA repair impairment

As miR-379-5p can significantly repress *PARP1* and *XRCC6*, which participate in DNA damage response and cell cycle control^39,40^, we further assess whether these processes are regulated by miR-379-5p.

**Fig. 3:**
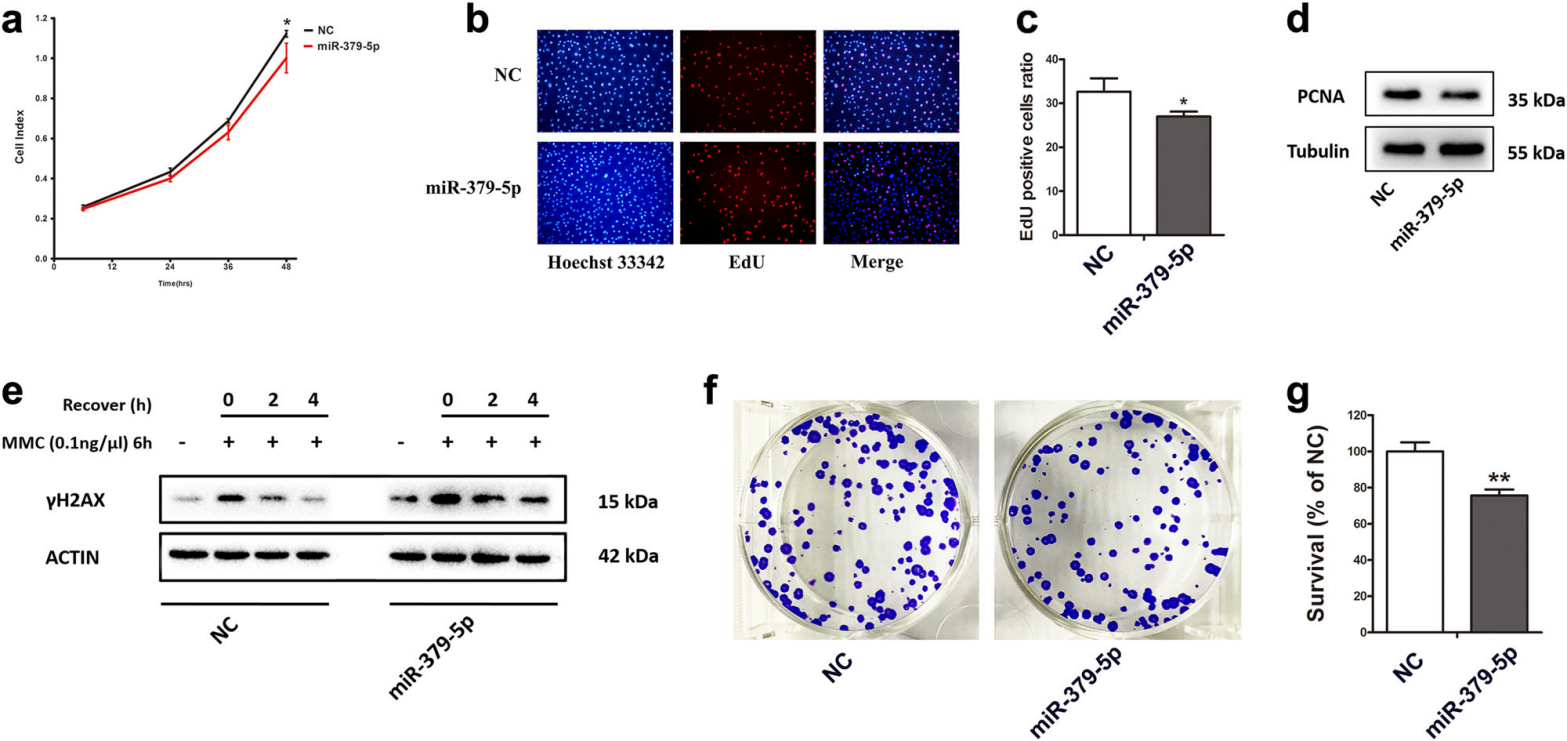
Overexpression of miR-379-5p leads to proliferation inhibition and DNA repair impairment **a** CCK-8 assay reveals that miR-379-5p overexpression led to suppression in cell proliferation. **b**,** c** EdU assay shows less EdU-positive cells in miR-379-5p overexpressed group. **d** PCNA level is much lower in KGN cells overexpressing miR-379-5p than negative control. **e** After exposed to MMC for 6 h, more of γH2AX is found in KGN cells overexpressing miR-379-5p. After recovery for 2 and 4 h, miR-379-5p delays the DNA damage repair. **f**,** g** The clonogenic survival of HeLa cells suffering MMC treatment and 2 h’ recovery. The clonogenic survival percent of miR-379-5p mimics group is significantly less than that of negative control. Asterisk (*) indicates a significant difference. *P < 0.05, **P < 0.01, two-tailed Student's *t* test

For cell proliferation analysis, Cell Counting Kit-8 (CCK-8) assays and 5-ethynyl-2′-deoxyuridine (EdU) staining were performed. Proliferating cell nuclear antigen (PCNA) was also detected by western blotting. CCK-8 assay revealed that overexpression of miR-379-5p led to an inhibition of proliferation in KGN cells (Fig. 3a). And, the EdU assay showed less EdU-positive cells in miR-379-5p overexpressed group (Fig. 3b, c). PCNA level was also much lower in KGN cells overexpressing miR-379-5p than negative control (Fig. 3d).

To illustrate the effect of miR-379-5p on DNA repair capacity, γH2AX, acting as a bio-marker for DNA damage, was detected to evaluate cellular response to DNA damage induced by mitomycin C (MMC). After exposed to MMC for 6 h, more γH2AX was found in KGN cells overexpressing miR-379-5p, implying higher sensitivity to MMC damage existed. When the DNA damage repair kinetics compared after recovery for 2 and 4 h, respectively, miR-379-5p delayed the DNA damage repair, which demonstrated that miR-379-5p adversely affected the progress of DNA breaks repair (Fig. 3e). HeLa cells clonogenic survival assay was conducted and showed that the clonogenic survival percentage of miR-379-5p mimics group was significantly less than that of negative control (Fig. 3f, g), indicating a harmful role of miR-379-5p in maintaining cell survival after DNA damage. Taken together, our results illustrated that forced expression of miR-379-5p led to inhibition of proliferation and impairment of DNA repair.

### *PARP1* and *XRCC6* are functional targets of miR-379-5p

**Fig. 4:**
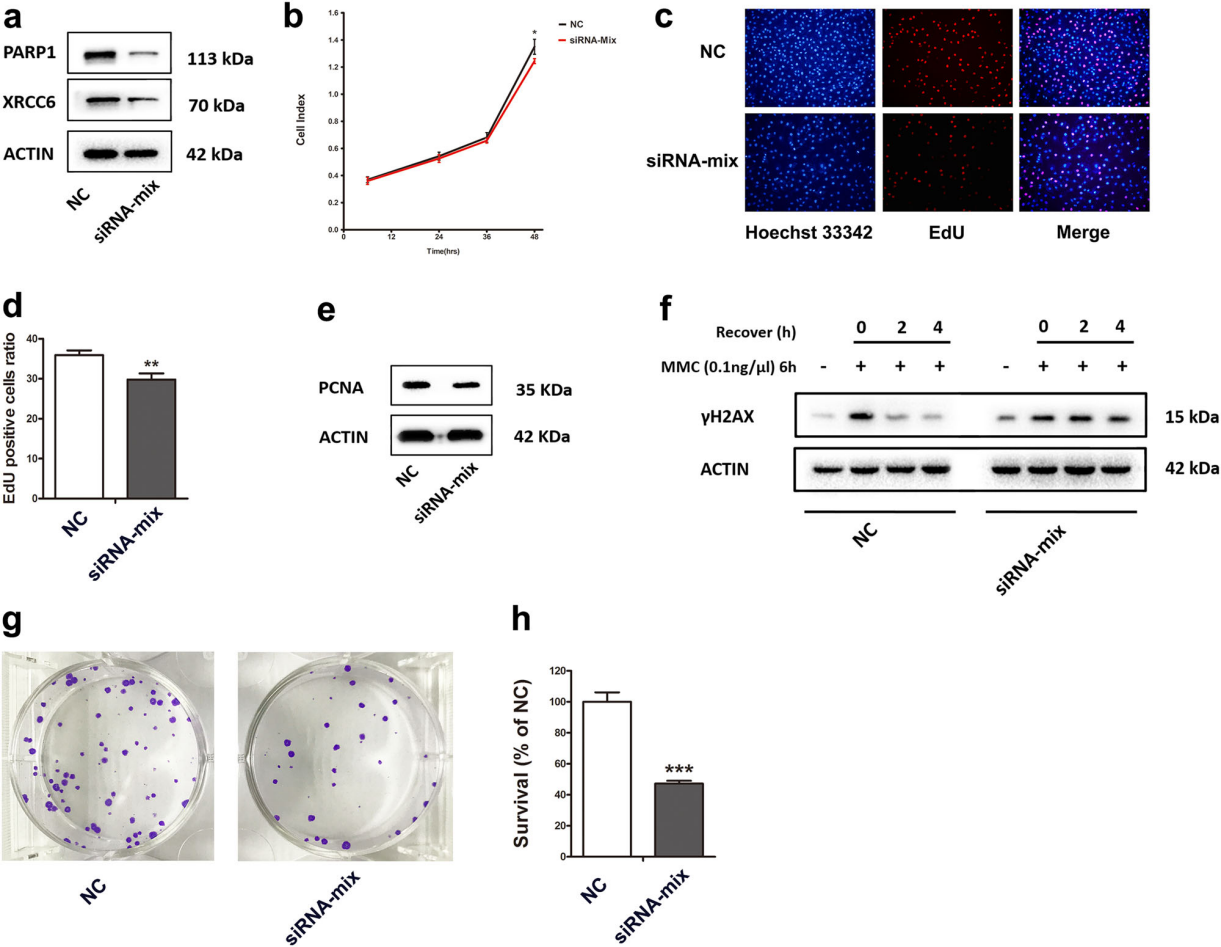
*PARP1* and *XRCC6* are functional targets of miR-379-5p **a** An efficient decrease in PARP1 and XRCC6 protein levels is confirmed by western blot in KGN cells 48 h post transfection. **b**,** c**,** d** Suppressed cell proliferation in *PARP1* and *XRCC6* knockdown group is indicated by CCK-8 assay (**b**) and EdU staining (**c**,** d**). **e** PCNA level is much lower in *PARP1* and *XRCC6* silencing cells than negative control cells. **f** After exposed to MMC for 6 h, and recovery for 2 and 4 h, *PARP1* and *XRCC6* silencing delayed the DNA damage repair. **g**,** h** The percent survival relative to the control is significantly lower in *PARP1* and *XRCC6* depletion cells. Asterisk (*) indicates a significant difference. *P < 0.05, **P < 0.01, ***P < 0.001, two-tailed Student's *t* test

To determine whether *PARP1* and *XRCC6* can mediate the observed biological function of miR-379-5p, we silenced *PARP1* and *XRCC6* in KGN cells via specific siRNAs. Western blotting confirmed an efficient decrease in the levels of PARP1 and XRCC6 protein in KGN cells 48 h post transfection (Fig. 4a). Then, CCK-8 assay and EdU staining were performed and revealed suppressed cell proliferation in *PARP1* and *XRCC6* knockdown group (Fig. 4b–d). Meanwhile, PCNA level was much lower in *PARP1* and *XRCC6* silencing cells than negative control cells (Fig. 4e).

Likewise, we examined the function of *PARP1* and *XRCC6* in DNA repair in vitro. After exposed to MMC for 6 h, and then recovery for 2 and 4 h, silencing of *PARP1* and *XRCC6* delayed the DNA damage repair (Fig. 4f). Coincident with the observation of clonogenic survival assay, we identified that lack of *PARP1* and *XRCC6* made HeLa cells more sensitive to DNA damage (Fig. 4g, h). The above results indicated that *PARP1* and *XRCC6* were required for protection of cells from DNA damage and efficient DNA damage repair.

### Reintroduction of *PARP1* and *XRCC6* abrogates the miR-379-5p-induced effects partially

**Fig. 5:**
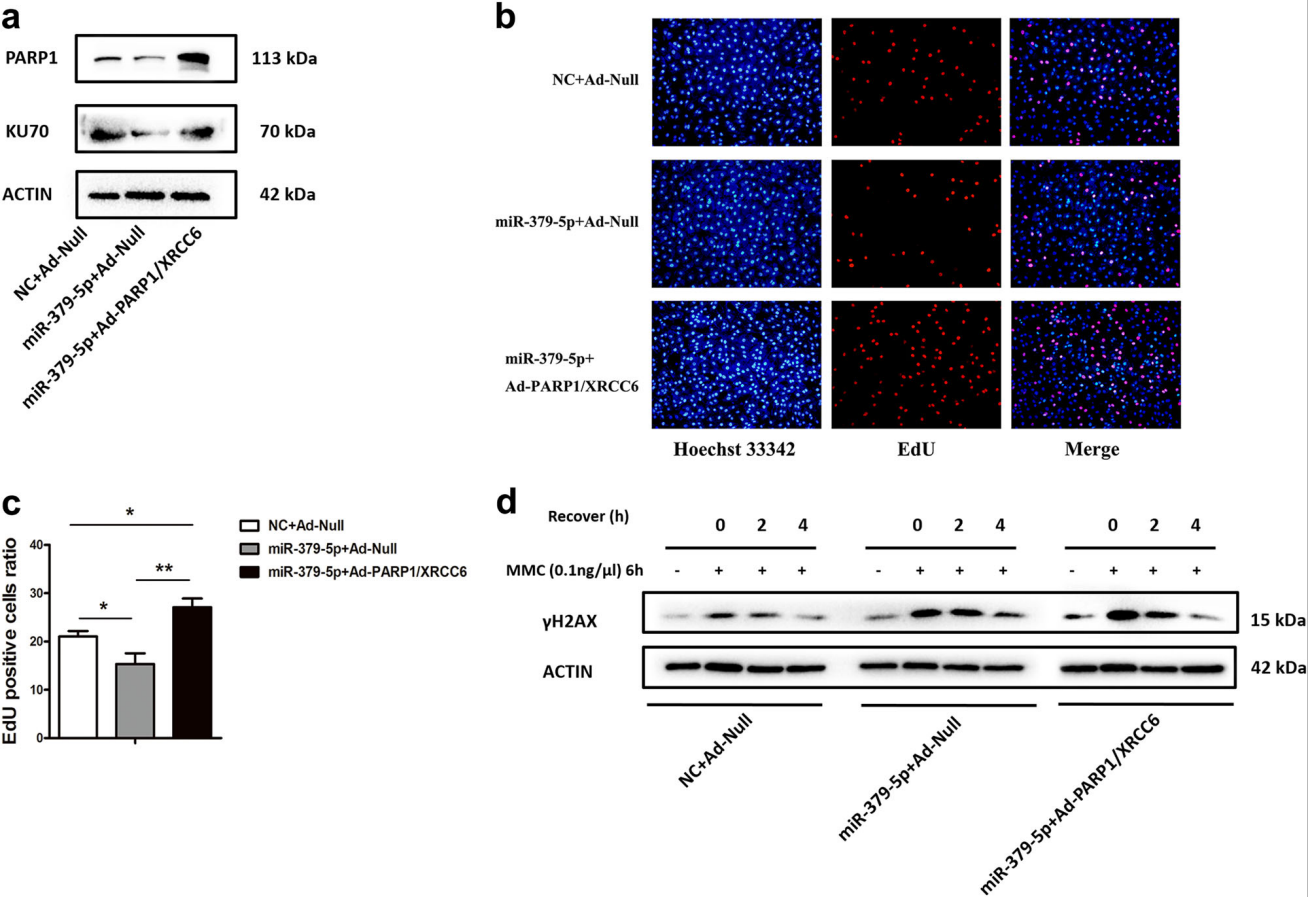
Reintroduction of *PARP1* and *XRCC6* abrogates the miR-379-5p-induced effects partially **a** An efficient increase in PARP1 and XRCC6 protein levels is confirmed by western blot in KGN cells 48 h post co-transfection infection. **b**,** c** *PARP1* and *XRCC6* overexpression could promote cell proliferation. **d** After exposed to MMC for 6 h, more of γH2AX is found in two groups overexpressing miR-379-5p. However, after 4 h recovery, *PARP1* and *XRCC6* overexpression group shows similarly less γH2AX as the NC + Ad-Null group. The results uncover that *PARP1* and *XRCC6* is required for efficient DNA damage repair process

To further verify the functional regulation between miR-379-5p and target genes, miR-379-5p mimic-transfected KGN cells were infected with recombinant adenovirus expressing *PARP1* and *XRCC6*. The infection efficiency was determined by western blotting (Fig. 5a). Interestingly, the roles of miR-379-5p on cell proliferation and DNA damage repair could be abolished to some extent, confirming the regulation of miR-379-5p on the two target genes (Fig. 5b–d).

## Discussion

In this study, two complementary microarray screenings were performed on granulosa cells derived from patients with biochemical POI for the first time, revealed miR-379-5p was expressed at higher levels in bPOI compared to normal controls. Further, in vitro experiments showed that miR-379-5p inhibited granulosa cell proliferation and sensitized GCs to DNA damage by repressing *PARP1* and *XRCC6*, which provide new etiologic explanation for POI.

Over the past few decades, hundreds of functional miRNAs have been identified and revealed perspectives for biological processes and diseases^14,15^. The relationships between miRNAs and POI have been investigated previously. Association studies evaluated that miRNA polymorphisms, such as miR-146aC>G, miR-196a2T>C, miR-499A>G, and miR-449bA>G, might be associated with high risk for POI^16,17^. Abnormal expression of miRNAs was observed in plasma or blood from POI patients, indicating that miRNAs serving as non-invasive diagnostic tools in clinic are promising^18–21^. However, uncertainties still existed owing to limited sample size and/or lacking functional evidences. Here our data significantly extended these findings by first uncovering a potentially pathogenic noncoding RNA, miR-379-5p, and two novel targets *PARP1* and *XRCC6* in GCs of bPOI patients. Creatively, in vitro experiments provided solid evidences to illustrate the role of miR-379-5p on granulosa cells function.

Normal function of granulosa cells is indispensable for reproduction and ovarian reserve^9,41,42^. Accumulated DNA damage and deficient DNA repair capacity in granulosa cells and oocytes may contribute to reproductive aging in mouse, rhesus monkey, as well as *homo sapiens*^43–45^. As aforementioned, perturbations in DNA repair genes, such as *MCM8* (OMIM 608187; POF10), *MCM9* (OMIM 610098), *CSB-PGBD3* (OMIM 609413; POF11), and *MSH5* (OMIM 603382; POF13), are responsible for POI in human^29–34^. In this study, decreased *PARP1* and *XRCC6* were observed in patients with bPOI. *PARP1* and *XRCC6* are pivotal genes involved in DNA damage repair. *PARP1* binds to the ends of DNA single-strand breaks and DNA double-strand breaks (DSBs) to facilitate DNA repair process^39,46–48^. *XRCC6* (also referred to as *Ku70*) cooperates with *Ku80* to form Ku heterodimer, which initiates nonhomologous end joining pathways in DSBs repair^40,49^. Recently, *PARP1* and *XRCC6* have been proved crucial for fundamental cellular processes, metabolism, ageing, and related diseases, such as cancers, diabetes, neurodegenerative, and cardiovascular diseases^39,46–49^. Both *Parp1*^*−/−*^ and *Xrcc6*^*−/−*^ mice exhibit symptoms of accelerated ageing^50–53^, however, little is known concerning *PARP1* and *XRCC6* with ovary ageing and female fecundity. With evidence of proliferation inhibition and DNA repair attenuation in GCs, *PARP1* and *XRCC6* were identified as direct targets and executives of miR-379-5p. Our results further corroborated the contribution of DNA repair in the etiology of POI, and a novel epigenetic explanation was brought up.

Roles of miRNAs expressed in ovarian tissues, such as follicular fluid, corpora lutea, and cumulus–oocyte complexes, have hitherto been evaluated^54–56^. However, the progress of finding key miRNAs in GCs from POI patients has been slow due to its poor collection. In contrast with overt POI, follicles have not yet been completely depleted at bPOI stage. Therefore, we collected the GCs from individuals with bPOI undergoing IVF-ET treatment. More importantly, it has been demonstrated a remarkably rapid decline of ovarian function after menses irregularity occurred, e.g., lasting 1–2 years at average^57^. Therefore, it is requisite to explore the phenotypes and pathogenesis in patients still at early stage of POI.

In summary, our study demonstrated altered profiles of miRNAs and mRNAs in the GCs from patients with bPOI. Functional studies further confirmed that upregulation of miR-379-5p suppressed GCs proliferation and adversely affected DNA repair function by negatively regulating expression of *PARP1* and *XRCC6*. This study provides new viewpoint for understanding the roles of noncoding RNA for GCs function, as well as potential etiologic mechanism for POI. Additional pathogenesis researches concentrated on miRNAs regulatory networks are warranted.

## Methods

### Human patient samples

The study was approved by the Institutional Review Board of Center for Reproductive Medicine, Shandong University, written informed consent was obtained from all participants. The clinical characteristics of bPOI and control women were shown in Table 1. Granulosa cells were isolated from each participant and stored at −80 ℃ until processed for RNA extraction as described previously^58^.

### MiRNA and mRNA expression profiling assays

The miRNA and mRNA expression profiling assays of the GCs from 20 participants (10 bPOI and 10 control women) were conducted by use of Exiqon miRCURY^TM^ LNA arrays (v18.0; contains 2043 capture probes covering all human miRNAs; Exiqon, Vedbaek, Denmark) and Arraystar Human LncRNA Microarrays (V3.0; covering a total of 26,109 protein-coding transcripts; Arraystar, Inc. Rockville, MD, USA), respectively. Microarray experiments and data analyses were performed by KangChen Bio-tech, Shanghai, China.

### RNA extraction and quantitative RT-PCR

Total RNA was extracted using the Trizol reagent (Takara Bio Inc., Dalian, China) by phenol–chloroform precipitation. MiRNAs were reverse transcribed individually by using miRNAs-specific reverse transcription primers and a mixture of dNTP (10 mM, Takara Bio Inc., Dalian, China), 5× M-MLV (Moloney Murine Leukemia Virus) buffer (10 mM, Takara Bio Inc., Dalian, China), RNase inhibitor (40 U/μl, Takara Bio Inc., Dalian, China) and M-MLV reverse transcriptase (200 U/μl, Takara Bio Inc., Dalian, China). While total RNA was reversely transcribed into cDNA using PrimeScript RT Reagent Kit with gDNA Eraser (Takara Bio Inc., Dalian, China) for genes validation. The real-time polymerase chain reactions were performed using LightCycler^®^ 480 SYBR Green I Master (La Roche Ltd, CH) and carried out by Roche LightCycle^®^ 480 (La Roche Ltd, CH). U6 RNA and GAPDH were used as endogenous controls for qPCR of miRNAs and mRNAs, respectively. Each sample was run in triplicate. Data were analyzed according to 2^−ΔΔCt^ method. The primers can be found in Supplementary Table 1.

### Cell culture and transfection

The KGN cell line (obtained from RIKEN BioResource Center, Ibaraki, Japan), a steroidogenic human granulosa-like tumor cell line^59^, was cultured in DMEM/F12 (HyClone, Logan, UT, USA) supplemented with 10% fetal bovine serum (FBS; Biological Industries (BioInd), Beit Haemek, Israel) and 1% antibiotics (HyClone, Logan, UT, USA). The human embryonic kidney (HEK) 293T cell line and HeLa (human cervix carcinoma cell line) cell line were grown in DMEM High Glucose (HyClone, Logan, UT, USA) supplemented with 10% FBS (BioInd, Beit Haemek, Israel) and 1% antibiotics (HyClone, Logan, UT, USA). All cells were cultured at 37 ℃ in a humidified atmosphere of 5% CO_2_ in air. MiR-379-5p mimics, specific siRNAs for *PARP1*/*XRCC6* and negative control, were designed (siRNAs for *PARP1* referred to Refs. ^60^ and ^61^) and synthesized by Genepharma Inc (Shanghai, China). MiR-379-5p mimics and siRNA-MIX were transfected at 50 nM and 100 nM, respectively, using X-tremeGENE siRNA Transfection Reagent (La Roche Ltd, CH). Sequences were provided in Supplementary Table 2.

### Recombinant adenoviruses

Adenoviruses were generated and purchased from VigeneBio (Shangdong, China). For in vitro experiments, cells were infected with 2 × 10^7^ plaque-forming units (pfu)/ml. Empty virus expressing only GFP served as control (Ad-Null).

### Protein extraction and western blot

Total protein was harvested in 1× SDS loading buffer and separated by sodium dodecyl sulfate polyacrylamide (SDS-PAGE) gel and electro-transferred to polyvinylidene fluoride membranes (Millipore, Billerica, MA, USA). After blocking with 5% milk, membranes were incubated with primary antibodies overnight at 4 ℃ and horseradish peroxidase-conjugated anti-rabbit or anti-mouse secondary antibodies for 1 h at room temperature. Immunoreactive bands were detected and analyzed with ChemiDoc MP Imaging System (BIO-RAD, Richmond, CA) and Image Lab Sofware. The following antibodies were used: anti-PARP1 (Cell Signaling Technology, 9532S, 1:1000), anti-XRCC6 (Abcam, ab3114, 1:250), anti-PCNA (Santa Cruz Biotechnology, sc-56, 1:1000), anti-γH2AX (Cell Signaling Technology, 9718s, 1:1000), anti-β-actin (Proteintech, 60008-1-Ig, 1:1000), anti-α-Tubulin (Proteintech, 66031-1-Ig, 1:1000), Peroxidase-conjugated Affinipure Goat Anti-Mouse (Proteintech, SA00001-1, 1:5000), Peroxidase-conjugated Affinipure Goat Anti-Rabbit (Proteintech, SA00001-2, 1:5000).

### Luciferase reporter assay

The luciferase reporter plasmid containing wild-type 3′-UTRs of *PARP1* and *XRCC6* were purchased from GeneCopoeia (Rockville, MD, USA). Mutagenesis was generated by site-directed mutagenesis (Quik Change Lightning Site Directed Mutagenesis Kit; Stratagene, LaJolla, CA) with the wild-type luciferase vectors as templates. Primers used were listed in Supplementary Table 2. Wild-type and mutated vectors were cotransfected with miR-379-5p mimics or negative control into HEK-293T cells using Lipofectamine 2000 (Invitrogen). Luciferase activities of cultured supernatant were measured 48 h later using Secrete-Pair^TM^ Dual Luminescence Kit (GeneCopoeia, Rockville, MD, USA) according to the manufacturer’s instructions.

### In vitro proliferation assays

KGN cells transfected or infected with miRNAs, siRNAs, or adenovirus for 24 h were reseeded in 96-well plates for different experiment purposes. Cell Counting Kit-8 (CCK-8; Beyotime, Jiangsu, China) assays were performed with ten replicates and Cell-Light^TM^ EdU DNA Cell Proliferation (EdU; Ribobio, Guangzhou, China) assays were carried out in triplicate according to the manufacturer’s instructions respectively.

### Mitomycin C sensitivity assay

KGN cells were seeded in 6-well plates and cultured 48 h after transfection or infection. Cells were harvested immediately or exposed to 0.1 ng/μl mitomycin C (MMC; Melonepharma) for 6 h to induce DNA damage, followed by harvested immediately or after recovery for 2 and 4 h in culture medium at 37 ℃. DNA damage marker γH2AX was detected in KGN cells with or without MMC treatment and recovery by western blot.

### Clonogenic survival

HeLa cells were cultured with siRNA-Mix of *PARP1/XRCC6* for 48 h. Then cells were exposed to 0.05 ng/μl mitomycin C (MMC; Melonepharma) for 6 h, followed by recovery for 2 h. The cells were harvested and counted, and reseeded into a new 6-well plate by 500 cells/well (three wells for duplicate) and incubated for 12–14 days. Colonies were stained with crystal violet (Beyotime, Jiangsu, China) and counted. Data were expressed as percent survival relative to the control: [(average treated count)/(average control count)]x100%.

### Statistical analysis

All statistical analyses were performed with the use of SPSS 21.0 (IBM) and GraphPad Prism 5. Data normality were assessed by Shapiro–Wilk’s test. Continuous data in normality distribution were presented as mean ± SD and determined with the two-tailed Student’s *t* test; otherwise data were expressed as median (IQR) and compared by two-tailed Mann–Whitney *U*-test. A *P* value of <0.05 was considered statistically significant. *P < 0.05, **P < 0.01, ***P < 0.001.

## Electronic supplementary material

Supplementary Tables
