# Supplementary material for: MicroRNA-379-5p is associated with biochemical premature ovarian insufficiency through PARP1 and XRCC6
Source: Cell Death Dis. 2018 Jan 24;9(2):106. doi: 10.1038/s41419-017-0163-8 (PMC5833760; doi:10.1038/s41419-017-0163-8)
Supplement: Supplementary file 1 — Supplementary Tables [file 41419_2017_163_MOESM1_ESM.docx]

**Supplementary Table 1. List of primers used for PCR assays and plasmids construction.**

|  | Primer Sequences |
| --- | --- |
| hsa-miR-379-5p-RT | 5’GTCGTATCCAGTGCGTGTCGTGGAGTCGGCAATTGCACTGGATACGACCCTACG3’ |
| hsa-miR-379-5p | F: 5’GGGGGGTGGTAGACTATGGAA3’  R: 5’GTGCGTGTCGTGGAGTCG3’ |
| U6-RT | 5’CGCTTCACGAATTTGCGTGTCAT3’ |
| U6 | F: 5’GCTTCGGCAGCACATATACTAAAAT3’  R: 5’CGCTTCACGAATTTGCGTGTCAT3’ |
| PARP1 | F: 5’CGGAGTCTTCGGATAAGCTCT3’  R: 5’TTTCCATCAAACATGGGCGAC3’ |
| XRCC6 | F: 5’GTTGATGCCTCCAAGGCTATG3’  R: 5’CCCCTTAAACTGGTCAAGCTCTA3’ |
| GAPDH | F: 5’GGGAAACTGTGGCGTGAT3’  R: 5’GAGTGGGTGTCGCTGTTGA3’ |
| PARP1-mut | F: 5’acttttgtcttccaaattaaaacacaaatatacttacccaagggct3'  R: 5’agcccttgggtaagtatatttgtgttttaatttggaagacaaaagt3' |
| XRCC6-mut | F: 5’ctcgacttatgtcgtcttcctagctcaggagaaac3'  R: 5’gtttctcctgagctaggaagacgacataagtcgag3' |

**Supplementary Table 2. List of miRNA mimics and siRNAs sequences used for transfection.**

| RNA oligos | Sense Sequences (5’-3’) |
| --- | --- |
| miR-379-5p mimics | UGGUAGACUAUGGAACGUAGG |
| PARP1-siRNA-1 | AAGCCUCCGCUCCUGAACAAU |
| PARP1-siRNA-2 | AAGAUAGAGCGUGAAGGCGAA |
| PARP1-siRNA-3 | AAGCCAUGGUGGAGUAUGATT |
| XRCC6-homo-203 | CAGGGUGGGAGUCAUAUUATT |
| XRCC6-homo-1164 | GGAGUCGUCAGAUUAUACUTT |
| XRCC6-homo-1726 | GGUUGAAGCAAUGAAUAAATT |
| Negative control | UUCUCCGAACGUGUCACGUTT |

Note: siRNAs for *PARP1* referred to reference 60.
